# New Approaches for Basophil Activation Tests Employing Dendrimeric Antigen–Silica Nanoparticle Composites

**DOI:** 10.3390/pharmaceutics16081039

**Published:** 2024-08-03

**Authors:** Silvia Calvo-Serrano, Esther Matamoros, Jose Antonio Céspedes, Rubén Fernández-Santamaría, Violeta Gil-Ocaña, Ezequiel Perez-Inestrosa, Cecilia Frecha, Maria I. Montañez, Yolanda Vida, Cristobalina Mayorga, Maria J. Torres

**Affiliations:** 1 Allergy Research Group, Instituto de Investigación Biomédica de Málaga y Plataforma en Nanomedicina—IBIMA Plataforma Bionand, Parque Tecnológico de Andalucía, 29590 Málaga, Spain; silcalser@gmail.com (S.C.-S.); jcespedeslagos13@gmail.com (J.A.C.); rubenfernandezsantamaria@gmail.com (R.F.-S.); frechacecilia@gmail.com (C.F.); lina.mayorga@ibima.eu (C.M.); mjtorresj@uma.es (M.J.T.); 2 RICORS Red de Enfermedades Inflamatorias (REI), 28029 Madrid, Spain; 3 Departamento de Medicina y Dermatología, Universidad de Málaga, 29071 Málaga, Spain; 4 Departamento de Química Orgánica, Universidad de Málaga, Campus Teatinos s/n, 29071 Málaga, Spain; esthermc@uma.es (E.M.); vgil51192@gmail.com (V.G.-O.); inestrosa@uma.es (E.P.-I.); 5 Instituto de Investigación Biomédica de Málaga y Plataforma en Nanomedicina—IBIMA Plataforma Bionand, Parque Tecnológico de Andalucía, 29590 Málaga, Spain; 6 Allergy Unit, Hospital Regional Universitario de Málaga-HRUM, 29010 Málaga, Spain

**Keywords:** PAMAM dendrimers, dendrimeric antigens, silica nanoparticles, drug allergy diagnosis, basophil activation test

## Abstract

In vitro cell activation through specific IgE bound to high-affinity receptors on the basophil surface is a widely used strategy for the evaluation of IgE-mediated immediate hypersensitivity reactions to betalactams. Cellular activation requires drug conjugation to a protein to form a large enough structure displaying a certain distance between haptens to allow the cross-linking of two IgE antibodies bound to the basophil’s surface, triggering their degranulation. However, no information about the size and composition of these conjugates is available. Routine in vitro diagnosis using the basophil activation test uses free amoxicillin, which is assumed to conjugate to a carrier present in blood. To standardize the methodology, we propose the use of well-controlled and defined nanomaterials functionalized with amoxicilloyl. Silica nanoparticles decorated with PAMAM–dendrimer–amoxicilloyl conjugates (NpDeAXO) of different sizes and amoxicilloyl densities (50–300 µmol amoxicilloyl/gram nanoparticle) have been prepared and chemically characterized. Two methods of synthesis were performed to ensure reproducibility and stability. Their functional effect on basophils was measured using an *in-house* basophil activation test (BAT) that determines CD63^+^ or CD203c^high^ activation markers. It was observed that NpDeAXO nanocomposites are not only able to specifically activate basophils but also do so in a more effective way than free amoxicillin, pointing to a translational potential diagnosis.

## 1. Introduction

Nanoscale science is an exciting scientific frontier that focuses on the length scale of approximately 1−100 nm. Our growing ability to create, manipulate, and characterize at the nanoscale is contributing to the exploration and exploitation of the biomedical applications of different nanomaterials and nanostructures. Nanoparticles (Nps) and dendrimers, as well as their combination in hybrid nanocomposites, are relevant to the present study, with potential in vivo and in vitro applications [1,2]. The interaction between these nanocomposites and the immune system is an area of interest which we apply to the diagnosis of allergic reactions to drugs [3,4] with the aim of dealing with the existing limitations of established methods.

Betalactam antibiotics (BLs) are drugs of first choice for the treatment of bacterial infections, but they are also the most common cause of allergic drug reactions, with an estimated prevalence of 10%. BL allergy is most commonly induced by immunoglobulin E (IgE)-mediated mechanisms, causing symptoms varying from simple skin involvement to anaphylactic shock [5,6].

The diagnosis of antibiotic allergy is complex, most times challenging, and, due to low sensitivity of the tests, usually overestimated. In fact, currently, only less than 30% of adults and 10% of children who report “allergy” to BLs are truly confirmed after an allergological work-up [7]. Moreover, whether truly allergic to the antibiotic or not, patients are banned from the drug for life, resorting to less effective second-line antibiotics, which are associated with a higher prevalence of infections by resistant bacteria, higher costs, longer hospitalizations, and increased readmission rates [7,8]; this is the reason why accurate diagnostic tests are needed.

BL allergy diagnosis involves clinical history, followed by in vivo methods consisting of a skin test (ST) and DPT (drug provocation test). Since clinical history may not always be accurate and the sensitivity of STs is not optimal, the final diagnosis usually relies on DPTs, which, due to patient risk, are contraindicated in severe reactions [9]. Given the described limitations of in vivo tests, in vitro tests can represent good alternatives for diagnosis. Indeed, the basophil activation test (BAT) and the quantification of serum-specific IgE (sIgE) are being used as complementary methods in combination with STs to avoid DPT despite their current suboptimal sensitivity [10,11,12].

The BAT is a flow-cytometry-based technique that has gained importance as a diagnostic tool for sIgE-mediated drug allergy [9] as it attempts to mimic in vivo IgE-mediated cell activation and mediator release during an allergic response. This test identifies basophils (anti-IgE, CCR3, CRTH2, and CD203c) and quantifies activation markers (CD63 and CD203c) expressed on their surface after stimulation with the suspected drug. BAT sensitivity depends on the drug involved; in the case of penicillins, it ranges from 22% to 55% [10]. If we focus on amoxicillin (AX), the most frequently consumed BL responsible for drug allergy, the sensitivity of the BAT is around 50%. Such a low sensitivity can be associated with the low level of sIgE on basophils, common in drug allergy and which limits its activation and consequent degranulation in the assay. Another limitation could lie in the nature of AX since it is a small molecule that cannot induce an allergic reaction alone. However, in blood, this drug reacts to proteins, forming covalent amoxicilloyl(AXO)–protein conjugates that feature both efficient antigen presentation and sufficient size to accomplish the minimal structural requirements for basophil activation [11]. Requirements refer to immunological recognition by at least two adjacent IgE antibodies that are bound to their high-affinity receptor (FcεRI) on the cell surface, which leads to the IgE cross-linking process and subsequent activation and degranulation with the release of inflammatory mediators that characterize an allergic reaction [13,14].

Research focused on understanding these triggering conjugates has described the identification of AX target proteins and modification sites, as well as their in vitro immunological recognition at the sIgE level [15,16], but none at the basophil level. As a consequence, the only thing we can assume about the BAT assay is that free AX binds to proteins in fresh blood samples. Although the nature and chemical composition of the conjugates that induce cell activation are not known and include inter-assay variability between subjects, it seems clear that the process is influenced by their size and multivalency of the drug–protein conjugates.

To gain insight into the structural requirements needed for optimal interaction between the drug moieties and sIgE on the basophil surface, we propose the use of nanoscale-size customized synthetic materials that can effectively interact with sIgE bound to receptors on the cell surface. Nanotechnology can handle existing problems with natural protein conjugates, such as low density of drug determinants and low reproducibility, and achieve effective diagnostic outcomes by controlling conjugate size and increasing the multivalence and the availability of peripheral drug antigenic determinants. Additionally, the use of homogeneous well-defined and characterized materials facilitates the standardization of the test and the interpretation of results.

We previously designed dendrimeric antigen (DeAn) conjugates, in which the dendrimer plays the role of a synthetic protein and is decorated with multiple units of drug antigenic determinant, with potential uses for sIgE quantification [17,18,19]. Another study demonstrated that DeAns participate effectively in the intramolecular cross-linking of sIgE bound to FcεRI on basophils [20]. Specifically, two-generation PAMAM dendrimers (G2 and G4) decorated with AXO resulted in DeAXO conjugates, which showed higher stimulation rates for the larger conjugates in BATs performed with samples of BL-allergic patients [20].

In line with these results, other studies focusing on the process of membrane antibody–receptor (IgE-FcεRI) binding and cross-linking reported that larger conjugates lead to higher degranulation degrees on rat basophilic leukemia cells [21,22]. Bidendron antigens (BiAns) based on multivalent AXO dendrons spaced by flexible polyethyleneglycol (PEG) linkers of different lengths (600–12,000 Da) showed, in the cellular assays sensitized with antibodies from sera of AX-allergic patients (AX-AP), activation responses only with the longer structures (containing PEG 6000–12,000 Da) involving an estimated distance of 13–20 nm between AXO determinants [22]. To achieve bigger nanomaterials, another study used gold Nps as a solid support for the covalent immobilization of a synthetic hapten ligand at high density, observing that 20 and 50 nm Nps elicited the greatest cellular response. However, a reduced hapten density inhibited degranulation [21]. Moreover, other parameters have been reported to influence efficient cross-linking, such as the density of ligand epitopes (or valency) [23], the proximity of the ligand epitopes and steric hindrance [24], three-dimensional structure, flexibility, or rigidity [13].

This study aims to develop nanomaterials to efficiently activate basophils in the context of AX allergy. Nanosystems were designed inspired by the optimal nano-size described for cell activation, as well as by previous studies of sIgE recognition of DeAns, and taking into account the advantages of Nps as platforms in bioapplications, such as robustness, high surface-to-volume ratio, size similar to biomolecules, and chemically tuneable surface. Silica Nps were chosen as an inert scaffold to increase the size of the DeAXO conjugates and to present the antigenic determinant disposed in a manner that allows simultaneous attachment to IgEs on cell receptors. In addition, the selection of silica was encouraged by our experience in the use of DeAn–silica particle composites (DeAn@SiO_2_; Φ = 500 nm) in immunoassays for the effective determination of sIgE from BLs from sample subjects [25,26].

Herein, different silica Nps decorated with DeAXO have been designed to evaluate the influence of both conjugate size and antigenic determinant density on the basophil activation mechanism. For this purpose, Nps of different sizes (20, 30, and 50 nm) have been prepared, and, by tuning the chemical modification protocols, different ligand (antigenic determinant) densities in their surface have been obtained. We further analyzed the effect of the different Nps and compared it with those produced by free AX in an *in-house* BAT based on a consensus protocol [27] in samples of AX-AP.

## 2. Materials and Methods

### 2.1. Nps Chemical Studies

Standard chemicals were obtained from Aldrich or VWR and used without further purification. PAMAM dendrimers were purchased from Aldrich. Milli-Q™ water was obtained by using the ultrapure Millipore^®^ Direct-Q^®^ 3. Phosphate-buffered saline (PBS) and 4 M lithium acetate buffer of pH 5.3 were prepared as described [28]. Macrosep^®^ Advance centrifugal devices with 100 K MWCO from Pall Lab were used for the Np dispersions.

#### 2.1.1. Np Synthesis

- **20dNps**

Silica particles were prepared adapting previously reported procedures [29,30]. Aqueous NH_3_ (25%, 0.75 mL) was added over a methanol/butanol (4:1) mixture (60 mL) and the mixture was stirred for 15 min. Then, tetraethyl orthosilicate (TEOS, 2.5 mmol) was added and the mixture was stirred at room temperature for 16 h to obtain the silica nanospheres. The particle dispersion was centrifuged (25 min, 3500 rpm; centrifugal devices with 100 K MWCO) and washed with HCl 1 M and Milli-Q™ water. A colloidal dispersion (300 mL) of Nps (20 nm of diameter; **20dNps**) in water was obtained.

- **30dNps**

Silica particles were prepared adapting previously reported procedures [29,30]. Absolute ethanol (500 mL) was added over a Tween-20/1-butylamine (1:1.1) mixture (2.1 g) and the mixture was stirred for 15 min. Then, tetraethyl orthosilicate (TEOS, 2.5 mmol) was added and the mixture was stirred at room temperature for 16 h to obtain the silica nanospheres. The particle dispersion was centrifuged (25 min, 3500 rpm; centrifugal devices with 100 K MWCO) and washed with HCl 1 M and Milli-Q™ water. A colloidal dispersion (300 mL) of Nps (30 nm of diameter; **30dNps**) in water was obtained.

- **50dNps**

Silica particles were prepared adapting previously reported procedures [31]. Aqueous NH_3_ (25%, 0.16 mL) was added over a water/ethanol (1:20) mixture (100 mL) and the mixture was stirred for 15 min. Then, tetraethyl orthosilicate (TEOS, 31.25 mmol) was added and the mixture was stirred at room temperature for 16 h to obtain the silica nanospheres. The particle dispersion was centrifuged (25 min, 3500 rpm; centrifugal devices with 100 K MWCO) and washed with HCl 1 M and Milli-Q™ water, and 300 mL of a colloidal dispersion of Nps (50 nm of diameter; **50dNps**) in water was obtained.

- **50Nps**

Silica particles were prepared adapting previously reported procedures [32]. Aqueous NH_3_ (25%, 7.12 mL) was added over ethanol (114 mL) and the mixture was stirred at 50 °C for 6 h. Then, tetraethyl orthosilicate (TEOS, 20 mmol) was added and the mixture was stirred at 30 °C for 6 days to obtain the silica nanospheres. The particles were centrifuged (40 min, 6500 rpm) and washed with HCl 1 M and Milli-Q™ water, and 800 mg of Nps (50 nm of diameter; **50Nps**) were obtained as a colorless solid after lyophilization.

#### 2.1.2. Np Surface Modification

- **20dNpNH_2_**, **30dNpNH_2_**, and **50dNpNH_2_**

A colloidal dispersion of Nps (**20dNps**, **30dNps**, and **50dNps**) in water (120 mL) was placed in a centrifugal device with 100 K MWCO. Toluene (120 mL × 3 times) was added and the mixture centrifuged (20 min, 6500 rpm) to change the solvent. Finally, toluene (300 mL) was added and the Nps dispersion was sonicated for 15 min. Then, 3-(aminopropyl)-triethoxysilane (APTES, 5 mmol) was added and mixture was sonicated for 1 h. The solution was refluxed under stirring overnight after the addition of 4.2 mmol of extra APTES. The particle dispersion was centrifuged (20 min, 6500 rpm; centrifugal devices with 100 K MWCO) and washed with toluene (×3 times) and Milli-Q™ water (×3 times). A colloidal dispersion of Nps in water was obtained.

- **50NpNH_2_**, **50Np^0.1^NH_2_** and **50Np^0.01^NH_2_**

**50Nps** were suspended in toluene (30 mL/100 mg of Nps) and sonicated for 15 min. Then, 3-(aminopropyl)-triethoxysilane (APTES) was added (see Supplementary Materials) and the mixture was sonicated for 1 h, after which it was refluxed under stirring overnight. The obtained particles were centrifuged (40 min, 6500 rpm), washed with toluene (×3 times) and Milli-Q™ water (×3 times), and finally lyophilized to obtain the desired amino-functionalized particles.

- **20dNpCO_2_H**, **30dNpCO_2_H**, and **50dNpCO_2_H**

A colloidal dispersion of Nps (**20dNpNH_2_**, **30dNpNH_2_** and **50dNpNH_2_**) in water (100 mL) was placed in a centrifugal device with 100 K MWCO. Dimethylformamide (DMF, 120 mL × 3 times) was added and the mixture centrifuged (20 min, 6500 rpm) to change the solvent. Finally, DMF (35 mL) was added and the Np dispersion was sonicated for 15 min. Then, succinic anhydride (8 mmol) in 15 mL of DMF was added to the previous mixture and sonicated for additional 15 min. Triethylamine (TEA, 0.8 mmol) was then added and the solution stirred at room temperature overnight. The particle dispersion was centrifuged (20 min, 6500 rpm; centrifugal devices with 100 K MWCO) and washed with DMF (×3 times) and Milli-Q™ water (×3 times). A colloidal dispersion of Nps in water was obtained.

- **50NpCO_2_H**, **50Np^0.1NH2^CO_2_H**, or **50Np^0.01NH2^CO_2_H**

Nps (**50NpNH_2_**, **50Np^0.1^NH_2_** and **50Np^0.01^NH_2_)** were suspended in DMF (2.5 mL/100 mg of Nps) and sonicated for 15 min. Then, succinic anhydride was added to the previous mixture and sonicated for an additional 15 min. Triethylamine (TEA) was then added and the solution stirred at room temperature overnight. The obtained particles were centrifuged (40 min, 6500 rpm), washed with DMF (×3 times) and Milli-Q™ water (×3 times), and finally lyophilized to obtain the desired carboxy-functionalized particles (see Supplementary Materials).

- **20dNpDe**, **30dNpDe**, and **50dNpDe**

A colloidal dispersion of Nps (**20dNpCO_2_H**, **30dNpCO_2_H**, and **50dNpCO_2_H)** in water (100 mL) was placed in a centrifugal device with 100 K MWCO. DMF (100 mL × 3 times) was added and the mixture centrifuged (20 min, 6500 rpm) to change the solvent. Finally, anhydrous DMF (100 mL) was added and the solution kept under argon pressure. Then, 1-(3-(dimethylamino) propyl)-3-ethylcarbodiimide hydrochloride (EDCI, 0.4 mmol) and *N*-hydroxisuccinimide (NHS, 0.7 mmol) in 10 mL of anhydrous DMF were added and the Np dispersion was sonicated for 15 min. Then, PAMAM-G2 dendrimers (1.7 × 10^−3^ mmol) in 10 mL of anhydrous DMF were added to the previous mixture and sonicated for an additional 15 min, after which the solution was stirred at room temperature overnight. The particle dispersion was centrifuged (20 min, 6500 rpm; centrifugal devices with 100 K MWCO) and washed with DMF (×3 times) and Milli-Q™ water (×3 times). A colloidal dispersion of Nps in water was obtained.

- **50NpDe**, **50Np^0.1^De**, **50Np^0.01^De**, **50Np^0.1NH2^De**, or **50Np^0.01NH2^De**

Nps (**50NpCO_2_H**, **50Np^0.1NH2^CO_2_H**, and **50Np^0.01NH2^CO_2_H)**, EDCI, and NHS were suspended in anhydrous DMF (1.5 mL/100 mg of Nps) and sonicated for 15 min. Then, PAMAM-G2 dendrimers in anhydrous DMF (1.5 mL/100 mg of Nps) were added to the previous mixture and sonicated for additional 15 min after which the solution was stirred at room temperature overnight. The obtained particles were centrifuged (40 min, 6500 rpm), washed with DMF (×3 times) and Milli-Q™ water (×3 times), and finally lyophilized to obtain the desired dendrimer-functionalized particles see Supplementary Materials).

- **20dNpDeAXO**, **30dNpDeAXO**, and **50dNpDeAXO**

A colloidal dispersion of Nps (**20dNpDe**, **30dNpDe**, and **50dNpDe**) in water (60 mL) was placed in a centrifugal device with 100 K MWCO. A 0.05 M Na_2_CO_3_/NaHCO_3_ aqueous buffer at pH 10.2 (60 mL × 3 times) was added and the mixture centrifuged (20 min, 6500 rpm) to change the solvent. Finally, 0.05 M Na_2_CO_3_/NaHCO_3_ aqueous buffer at pH 10.2 (60 mL) was added and the Np dispersion was sonicated for 15 min. Then, 4 mL of a freshly prepared solution of amoxicillin (AX) 10 mg/mL in 0.05 M Na_2_CO_3_/NaHCO_3_ aqueous buffer at pH 10.2 was added to the previous mixture and sonicated for an additional 15 min, after which the suspension was stirred at 4 °C for 7 days. During this period, 10 mg of AX was added at approximately 24 h intervals. The particle dispersion was centrifuged (20 min, 6500 rpm; centrifugal devices with 100 K MWCO) and washed with PBS (×3 times) and Milli-Q™ water (×3 times). A colloidal dispersion of Nps in water was obtained.

- **50NpDeAXO**, **50Np^0.1De^DeAXO**, **50Np^0.01De^DeAXO**, **50Np^0.1NH2^DeAXO**, or **50Np^0.01NH2^DeAXO**

Nps were treated following previously reported procedures [25,26] to obtain the desired functionalized particles.

#### 2.1.3. Np Characterization

Dynamics light scattering (DLS)

The hydrodynamic diameter of the particles has been determined following previously reported procedures [25,26] using a Malvern Zetasizer Nano ZS90 instrument (“red laser” (λ = 633 nm) with a detection angle of 90°).

Transmission electron microscopy (TEM)

TEM images were recorded following previously reported procedures [25,26] in a PHILIPS CM-200 instrument.

Z Potential measurements

Z potential measurements were carried out following previously reported procedures [25,26] in a Malvern Zetasizer Nano ZS90 instrument.

Fourier-transformed infrared (FTIR) spectroscopy

FTIR measurements were carried out following previously reported procedures [25,26] in a Nicolet Nexus spectrometer (Thermo Fisher Scientific, Waltham, USA) with a Smart Golden Gate attenuated total reflectance (ATR) accessory.

Quantification of free primary amino groups

The quantification of free primary amino groups on the Np surface was carried out following a previously described procedure [25,33] using a VARIAN CARY 100BIO UV-Visible spectrophotometer. For Nps in dispersion, 1 mL of the corresponding colloidal dispersion was dried and weighed. This solid residue was dispersed in 1 mL of Milli-Q™ water and the suspension used in the test. Values were normalized to the obtained Np mass. For solid Nps, 1 mL of the corresponding Np suspension (1.5mg Nps/mL Milli-Q™ water) was used.

^1^H-NMR spectra of **50NpDeAXO**

^1^H HRMAS-NMR (High-Resolution Magic Angle Spinning Nuclear Magnetic Resonance) spectra were recorded at room temperature in an AVANCEIII HD 600 (Bruker AXS, Billerica, USA) spectrometer using a double-resonance probe of 4 mm at a spinning rate of 5 kHz. The magnetic field was 14.1 T corresponding to a ^1^H resonance frequency of 600.09 MHz. The ^1^H chemical shifts are referenced to D_2_O. ^1^H HRMAS-NMR spectra were recorded with a 4.6 us 90° pulse and 5 s delay and summing 300 scans.

### 2.2. Patients Selection and Allergological Work Up

Patients with a clinical history of immediate hypersensitivity reactions to AX (AX-APs) and tolerant healthy controls (HCs) were diagnosed according to European Academy of Allergy and Clinical Immunology guidelines [10,34] with a detailed clinical history, followed by a positive STs (SPT and/or IDT), and if negative, DPT with AX. Patients were classified according to the severity of the reactions as grade I (urticaria/angioedema), grade II (anaphylaxis), grade III (shock), and grade IV (ARREST) following the Ring and Messmer classification [35].

A skin prick test (SPT) and, if negative, intradermal test (IDT) were carried out as previously described [34] using solutions prepared daily from DAP Penicillin^®^ test Kit (Diater S.A, Madrid, Spain) at maximum concentrations of 0.04 and 0.5 mg/mL of major (benzylpenicilloyl-octa-L-lysine (BP-OL) and minor determinants (sodium benzylpenilloate), respectively, and with 20 mg/mL for AX and for clavulanic acid. Readings were taken after 20 min. If the ST was negative, DPT was performed firstly with penicillin V (Laboratorios ERN S.A., Barcelona, Spain) and, if negative, a second time with AX (GlaxoSmithKline, Madrid, Spain) [34]. DPT was developed by oral route in a single-blind placebo-controlled procedure at incremental doses, starting with lower doses and with a minimum 30 min interval between each until the total cumulative therapeutic dose [36]. Patients were monitored during DPT and for 2 h after the last dose.

### 2.3. Cell Viability Measurement

Basophil viability after stimulation with the different Nps was assessed using 7-aminoactinomycin D (7-AAD, (Invitrogen, Carlsbad, CA, USA), which is passively taken up by cells with loss of membrane integrity, like non-viable basophils. Briefly, cells were incubated with the different Nps for 25 min, lysed, washed, and incubated with 7-AAD. The percentage of cell viability was recorded in the region of low 7-AAD signal, in the basophil gate (CCR3^+^/CD203c^+^), with a minimum acquisition of 500 basophils per sample. The following conditions were compared: no stimulus, anti-IgE, N-Formylmethionyl-leucyl-phenylalanine (fMLP), AX, and Nps.

### 2.4. Basophil Activation Test (BAT) with Different Nanoparticles (Nps)

An *in-house* BAT based on a recent consensus protocol was performed [27] on AX-APs and HCs. The drug-containing stimulus solutions, either the free AX or Nps that have been preserved in the solid state, were prepared just prior to the assay. In the case of Nps prepared as suspension, this fresh preparation was not possible. Briefly, heparinized blood samples were exposed to 100 µL of 2.5, 1.25, and 0.25 mg/mL AX (Alpha Aesar, Heysham, UK) [27] or **NpDeAXO** at a concentration range of 0.005 to 300 µM of AXO units, depending to the Np used. Anti-human IgE (Becton-Dickinson, East Rutherford, New Jersey, USA) antibody at 0.01 mg/mL and fMLP at 4 µM were used as positive controls, while stimulation buffer was used as negative control. Then, basophils were stained with APC-anti-human CCR3, FITC-anti-human CD63, and PE-anti-human CD203c monoclonal antibodies (Biolegend, San Diego, CA, USA). Stained cells were analyzed by FACSCalibur flow-cytometer (Becton-Dickinson Bioscience, San Jose, CA, USA) by acquiring more than 500 basophils per sample, identified as SSC^low^/CCR3^+^ [37]. Their level of activation was analyzed by the expression of CD63 or the upregulation of CD203c (CD203c^high^) markers using FlowJo^®^ software v.8.2 (FlowJo LLC, Becton Dickinson, Ashland, OR, USA).

### 2.5. Statistical Analyses

Normality was assessed by the Kolmogorov–Smirnov test. Quantitative variables without normal distribution were compared with Mann–Whitney U and Kruskall–Wallis tests and qualitative variables with X^2^ test. Receiver operating characteristic (ROC) curves were performed to calculate the cut-offs for each of the NPs. Figures were made with GraphPad Prism 7 (GraphPad Software Inc, San Diego, CA, USA) and statistical analyses were performed using the SPSS program version 25.

## 3. Results and Discussion

### 3.1. Synthesis and Characterization of Nps

A series of SiO_2_ Nps of different sizes ranging from 20 to 50 nm were prepared and characterized. All Nps were prepared using the Stöber methodology [38,39]. However, it has been described that the preparation of Nps with a uniform size of below 50 nm is rather difficult. Thus, the water-in-oil microemulsion process was adapted depending on the desired final particle size to obtain **20dNps**, **30dNps**, and **50dNps** [39]. Based on reported methodologies [29,30], the molar ratio of TEOS/aqueous NH_3_ (25%)/organic solvent was modified until observing the desired size NPs. Nanoparticle size and distribution were determined by dynamic light scattering (DLS) experiments, and those were consistent with the observed TEM images (Figures S1 and S2, Supplementary Materials). From the TEM images, the diameter of the particles is about 20, 30, and 50 nm (**20dNps**, **30dNps**, **50dNps**, respectively). The average particle diameters in solution determined by DSL are 28, 37, and 52 nm, respectively. Those values agree with the TEM observation and indicate a rather good monodispersity of the particles. However, thermodynamically, Nps tend to agglomerate to reduce high surface energy, a process that occurs more frequently in the case of smaller Nps [39]. As a result, we observed that **20dNps**, **30dNps**, and **50dNps** are sufficiently stable in diluted solution but, when centrifuged to obtain a solid (or a concentrated solution), they agglomerate and do not disperse well again in solution, forming aggregates of various sizes that cannot be controlled. This makes the manipulation of the Nps difficult for the chemical modification of their surface, the consequent chemical characterization, and the performance of diagnostic tests. Moreover, it is a major handicap for their application in hospitals.

Therefore, great effort was invested to obtain Nps of these sizes which could be handled in the solid state and, when added to aqueous media, would disperse easily without forming aggregates. Adapting previously reported procedures [32], **50Nps** were obtained as a colorless solid, easy to manipulate and easily dispersible in aqueous media. From the TEM images, we can observe that the prepared nanoparticles were highly monodispersed spheres about 50 nm in outer diameter. The average particle diameter in solution determined by DSL is 60 nm (Figures S1 and S2, Supplementary Materials).

**Figure 1:**
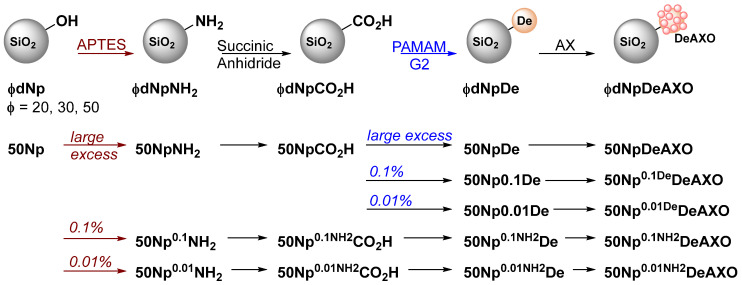
General procedure for the chemical modification of ϕ**dNp** dispersions and **50Nps** with different DeAXO densities in their surface.

For the covalent immobilization of the dendrimeric antigen (DeAXO) on the Np surface, we used our previously described procedure (Figure 1) [25,26]. First, Nps were treated with APTES to introduce amino terminal groups to the particles surface. These groups were then treated with succinic anhydride to attach the carboxylic moiety. Generation-2 PAMAM dendrimers were covalently anchored through the formation of an amide bond between carboxylic moiety on the particle surface and the amino terminal groups of the PAMAM dendrimers. Finally, AX was reacted with the free amino groups of the dendrimers to generate the dendrimeric antigen DeAXO on the Np surfaces through the opening of the antibiotic β-lactam ring.

As previously mentioned, the chemical functionalization of **20dNps**, **30dNps**, and **50dNps** was carried out always maintaining low concentration dispersions of Nps. For this purpose, centrifugal devices with a 100 K cut-off filtration membrane were used, allowing us to exchange solvents for the different reactions as well as to purify the obtained modified Np dispersions. A large excess of the reagents was always used to achieve the maximum surface modification at all times.

The obtained Nps dispersions were analyzed with Zeta potential (ξ) measurements and evaluations of the amount of free primary amino groups present on the Np surface using a ninhydrin test (see Table S1, Supplementary Materials). Zeta potential (ξ) measurements were used to monitor the chemical modifications performed on the Np surfaces, since modification of their chemical nature was expected to alter its charge. The ξ values obtained from PBS dispersions (solvent that mimics physiological pH 7.4) agree with the expected surface charge [25,26]. Negative values were observed for Nps with negatively charged terminal functional groups in PBS (-OH for **ϕdNp** or -COOH for **ϕdNpCO_2_H**, see Figure 1). On the other hand, ξ positive values have been observed for Nps where positively charged groups are present (-NH_2_ for **ϕdNpNH_2_** and **ϕdNpDe**). It should be noted that the values observed for Nps with dendrimers (**ϕdNpDe**) practically double those observed for **ϕdNpNH_2_**, and this is is consistent with the introduction of 15 amino groups per dendrimer [33]. The reaction with AX caused a shift in the using of the zeta potential to values around ξ = −8 mV for **ϕdNpDeAXO**, which is in perfect agreement with the data obtained in our previous works [25,26].

Ninhydrin test values are very useful to evaluate the degree of amino functionalization of the Nps (see Table S1, Supplementary Materials). An almost complete coverage of the particle surfaces with the dendrimers has been observed [25,26]. Considering that all the amino groups of the dendrimers reacted [17], we can then assume that the amounts of AXO units are approximately 290 µmol per gram of **20dNpDeAXO** and 270 µmol per gram of **30dNpDeAXO** or **30dNpDeAXO** (Table 1). As expected, the smallest particles have the highest number of AXO groups per gram since they have the highest surface area/mass ratio. No significant differences were found into the amount of AXO/gram between Nps of 30 and 50 nm of diameter.

The chemical functionalization of **50Nps** was carried out following our previously described procedures working with the solid dried Nps [25,26]. In a first modification, a large excess of the reagents was always used to achieve the maximum surface modification, obtaining **50NpDeAXO**. Two approaches have been used to achieve different DeAXO densities on the particle surface in a reproducible way. In the first one, the amount of APTES used in the first step of the chemical procedure was reduced 0.1% and 0.01% to obtain **50Np0.1NH_2_** and **50Np0.01NH_2_**, respectively (marked in red in Figure 1). In the second one, the amount of PAMAM-G2 dendrimers added was reduced by the same proportion to obtain **50Np0.1De** and **50Np0.01De**, respectively (marked in blue in Figure 1). Thus, four more different Nps decorated with dendrimers on their surface have been obtained, which, after AX treatment, have given rise to Nps with different degrees of surface functionalization.

Zeta potential (ξ) values show the same trend as in the case of the previous Nps and are consistent with the chemical modifications that the Nps undergo on their surface throughout the process (see Table S2, Supplementary Materials). In this case, the estimation of the degree of functionalization of the Nps and therefore of the DeAXO density on their surface is determined by the values obtained from the ninhydrin test. The number of AXO units that we can assume were incorporated into the Nps, expressed as µmol of AXO per gram of Nps, are shown in Table 1

**Figure 2:**
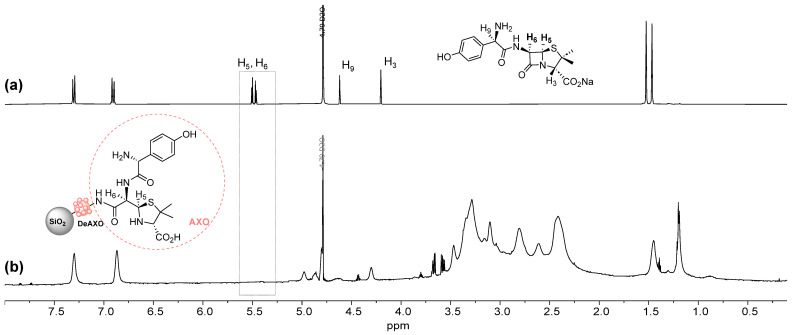
NMR spectra of (**a**) AX in basic D_2_O and (**b**) **50NpDeAXO** in D_2_O suspensions.

^1^H-NMR spectra of **50NpDeAXO** were recorded (Figure 2). No signals are observed around 5.5 ppm, indicating the absence of closed β-lactam rings. The rest of the signals confirm the presence of the PAMAM dendrimers (between 3.7 and 2.0 ppm) and the AXO units (between 6.75 and 7.5 ppm) in the Nps [19].

Stability of **50NpDeAXO** (stored as solids) were checked with DLS measurements of freshly prepared dispersions in distilled water at different time-points (0, 1, 2, 3, 4 and 5 h after preparation, more than enough time to perform the BAT assay). The results of DLS showed no aggregates. This is an indication that the preparation of dispersions without aggregates is feasible after resuspension of the stored Nps (as a solid at 5 °C, at least for a 6-month period of storage) (Figures S2–S7 in Supplementary Materials).

### 3.2. Evaluation of Basophil Activation Capacity of Nanoparticles: **20dNpDeAXO**, **30dNpDeAXO**, **50dNpDeAXO**, and **50NpDeAXO**

**Figure 3:**
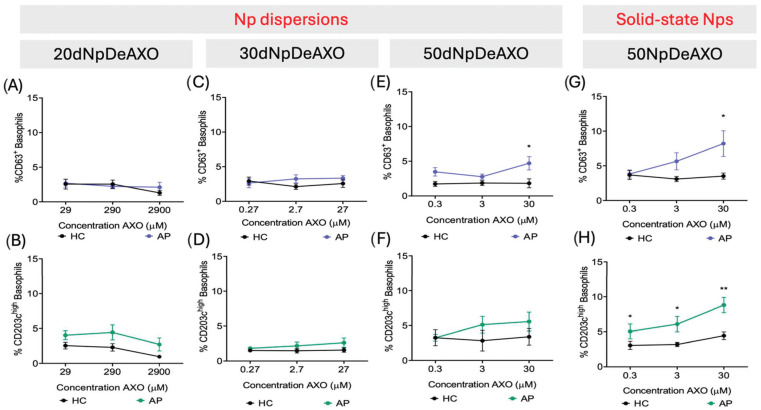
Basophil activation test (BAT) dose–response curves of **NpDeAXO** of different sizes: 20 nm (**A**,**B**), 30 nm (**C**,**D**), and 50 nm (**E**,**F**) and (**G**,**H**). *Np dispersions* or *solid-state Nps* labels at the top of the figure only indicate the synthetic methodology used for Np preparation. Black lines represent healthy controls (HCs), and blue and green lines represent allergic patients (APs). Size sample included HCs (N = 10) and APs (N = 10) for Nps synthesized as dispersions (**A**–**F**), while HCs (N = 45) and APs (N = 54) were included in the study for the Nps synthesized as a solid state. * *p* < 0.05, ** *p* < 0.01.

DeAXO-functionalized Nps obtained as colloidal dispersions, including those of 20nm (**20dNpDeAXO)**, 30nm (**30dNpDeAXO)**, and 50 nm (**50dNpDeAXO)**, were evaluated for their ability to activate basophils by BAT in samples from 10 AX-APs and 10 HCs. Clinical features of the AX-AP cohort can be found in Table S3. Results indicate that **20dNpDeAXO** and **30dNpDeAXO** did not produce any remarkable basophil activation (below 5%) in AX-APs or HCs at all concentrations used, measured either by CD63 or CD203c^high^ activation markers (Figure 3A–D). Interestingly, **50dNpDeAXO** triggered basophil activation, showing a higher percentage of CD63^+^basophils in AX-APs compared to HCs (Figure 3E). Conversely, when using the CD203c^high^ marker, despite a dose–response activation, discrimination between AX-APs and HCs was not significant (Figure 3F).

To perform more accurate studies about the effect on basophils of DeAXO-decorated Nps, we prepared Nps with an improved methodology to isolate them in a solid state (**50NpDeAXO**), which allows a more precise control of the DeAXO display compared to Nps obtained as dispersions and offers an increased reproducibility in the preparation of dispersions for the BAT assay and consequently in its result. In addition, in view of a potential use of the **50NpDeAXO** as a platform for downstream applications involving whole blood, toxicity must be excluded. Therefore, the viability of basophils treated with **50NpDeAXO** was investigated by incubating samples with the different AX or Np concentrations, followed by 7-AAD viability staining. We observed high levels of viability with **50NpDeAXO**, like the ones obtained with free AX (Figure S8).

The selected **50NpDeAXO**, because of the optimal Np size, 50 nm, and the improved synthetic method, which allows for greater stability and reproducibility in the measurements, were evaluated for their ability to activate basophils, by BAT, in samples from 54 AX-APs and 45 HCs. Further on, although both 50nm Nps (**50dNpDeAXO** and **50NpDeAXO)** allowed a clear discrimination of AX-APs and HCs, comparative analysis revealed that **50NpDeAXO**, at 30 µM, showed a higher ability to activate basophils in AX-APs, either through CD63 or CD203c^high^ activation markers (8.2% and 8.8%, respectively) compared to **50dNpDeAXO** (4.7% and 5.6%, respectively; Figure 3E–H).

A different sample size has been evaluated with each of the Nps of the same 50 nm size, dispersed or in solid state, which may partly explain the difference in the results between Figure 3E,G. However, being able to more accurately measure/weigh the amount of AXO units supported on the Nps put into the assay when using **50NpDeAXO**, compared to dispersed **50dNpDeAXO**, should imply a higher reproducibility and precision in the results obtained and may mainly explain these improved results obtained with **50NpDeAXO**. The later results confirmed that controlling the exact amount of amoxicilloyl supported in the Nps that are evaluated in the cell assay is very important to obtain accurate results. In fact, when we compare the two synthesis methods, we observe that obtaining particles of the same size (50 nm) in a more controlled manner also improves and provides better results. The evaluation of precise **NpDeAn** in the BAT, in which the size of the Nps is crucial for activation, indicated, due to controlled nano-size, the optimal distance of 50 nm for obtaining maximal activation in this assay. These findings underscore the importance of considering the Np size and synthesis method in the design of nanomaterials for biomedical applications, at least in the allergy field.

In agreement with these results, other studies on the cell activation process via membrane antibody–receptor (IgE-FcεRI) binding and cross-linking have described that larger synthetic immunogens lead to higher degranulation degrees on rat basophilic leukemia cells [21,22]. Flexible BiAns based on multivalent AXO dendrons showed activation responses only with the longer structures presenting an estimated distance of 13–20 nm between AXO determinants [22]. Another study involving rigid supports, as herein, reports that gold Nps fully decorated with a synthetic hapten ligand of sizes larger than 19.8 nm and up to 50 nm exhibit more efficient cell activation [21].

### 3.3. Effect of AXO Density on the Np Basophil Activation Capacity

**Figure 4:**
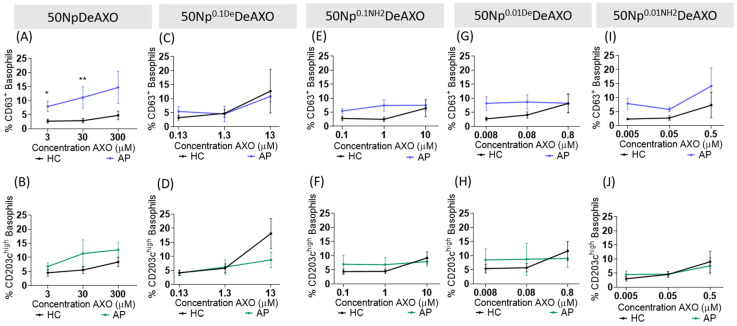
BAT dose–response curves of Nps with different DeAXO surface densities: 300 µmol AXO/gNp (**A**,**B**); 130 µmolAXO/gNp (**C**,**D**); 100 µmol AXO/gNp (**E**,**F**); 80 µmol AXO/gNp (**G**,**H**); 50 µmol AXO/gNp (**I**,**J**). Black lines represent healthy controls (HCs), and blue and green lines represent allergic patients (APs). Size sample included HCs (N = 6) and APs (N = 4) (**A**–**J**). * *p* < 0.05, ** *p* < 0.01.

Given the substantial potential of DeAXO-decorated Nps, it was critical to undertake a comprehensive study of the influence of the degree of functionalization with AXO on the Nps to activate basophils from AX-APs in a specific way (Figure 4). For this purpose, a series of **50NpDeAXO** with different surface DeAXO densities was obtained, namely **50NpDeAXO**, **50Np^0.1De^DeAXO**, **50Np^0.1NH2^DeAXO**, **50Np^0.01De^DeAXO**, and **50Np^0.01NH2^DeAXO** containing between 300 and 50µmolAXO/gNp (Figure 1 and Table 1). The capacity of all these **50NpDeAXO** derivatives to activate basophils was tested in AX-APs (N = 4) and HCs (N = 6).

BAT dose–response curves were constructed to evaluate the relationship between the concentration of Np-surface-displayed AXO and immune activation (Figure 4). Of all Nps tested, **50NpDeAXO** (300 μmol AXO/g Np) was the only one that induced basophil activation in a dose-dependent manner (Figure 4A,B). Consistently, Nps with the higher AXO content on their surface exhibited an enhanced discriminatory capacity between AX-APs and HCs (*p* = 0.019 and 0.0095, at 3 µM and 30 µM, respectively) when evaluating the expression of CD63 activation markers (Figure 4A).

In contrast, the rest of the Nps, displaying lower densities of AXO, showed null capacity to discriminate AX-APs from HCs (Figure 4C–J). Interestingly, the different treatments of the Nps to chemically modify them in order to have less DeAXO coating did not seem to influence their efficacy through any of the activation markers tested (CD63 or CD203c^high^) (Figure 4E,F,I,J).

For this BAT application, allergens may contain at least two epitopes and the distance between epitopes is crucial for activation. Nps with the higher surface coverage of DeAn seem particularly efficient in cross-linking sIgE on basophils. By decreasing the AXO surface coverage, activation decreases, probably due to increased spacing between each AXO molecule.

It is likely that the multivalency of the dendrimers in NpDeAXO favors the IgE interaction via a dendritic or synergistic effect and thus degranulation. This is consistent with cell activation induced by other multivalent systems: bigger dendrimer-derived structures [20,22,23] or Np rigid systems displaying the higher valence of ligands (antigenic determinants) induced increased degranulation in antigen-study interactions [21]. However, a reduced hapten density inhibited degranulation [21].

To sum up, the biggest Nps with the highest density of AXO were the best performer in terms of patient basophil activation, most probably through a specific AXO recognition. However, as it can be seen in Figure 4A, at the maximum concentration of Nps in the assay, expressed in terms of an AXO unit concentration as 300 µM, the cellular response was highly variable, resulting in a high deviation. We believe that the dispersion at this high concentration of Nps is relatively unstable; furthermore, it is difficult for the operator to handle and we cannot assume that the dispersions tested are always homogeneous. Consequently, dispersions obtained at such high Np concentrations influence the variation in the BAT results.

### 3.4. AXO Displayed in Nps is more Potent Than Free AX in Inducing Specific Basophil Activation

**Figure 5:**
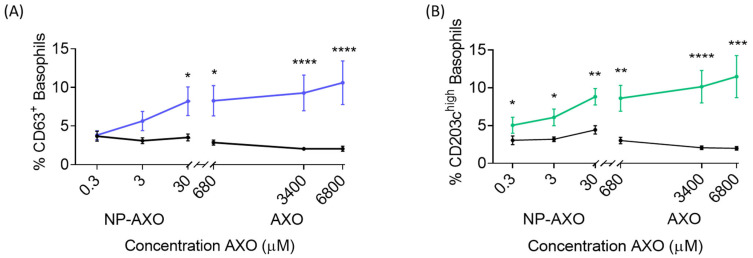
BAT dose–response curves using **50NpDeAXO** and free AX at three different concentrations. APs (N = 54) are depicted by blue or green lines and HCs (N = 45) are depicted by black lines. * *p* < 0.05, ** *p* < 0.01, *** *p* < 0.001, **** *p* < 0.0001.

We wanted to compare the efficiency of sIgE bridging depending on the way AX is presented, free AX or at the surface of the Nps, by analyzing the basophil activation levels in terms of AX concentrations (Figure 5). Due to the intrinsic characteristic of insolubility of the nanoparticles, they could not be evaluated at the same high concentration as the free AX. It was only possible to evaluate up to the concentration of the Np dispersions obtained that were stable. In fact, the Np concentration of 300 µM of AXO was discarded because in cytometry it generated a lot of debris and hindered the selection of the study population and a correct analysis. Unfortunately, Np concentration could not be increased beyond 30 µM AXO due to a compromise of Np solubility that impaired flow-cytometer data acquisition (Supplementary Figure S9). Samples from 54 AX-APs and 45 HCs were analyzed by BAT using free AX at 6800 µM, 3400 µM, and 680 µM and **50NpDeAXO** at 30 µM, 3 µM, and 0.3 µM of AXO, represented in Figure 5. Data showed that **50NpDeAXO** at the highest concentration of AXO (30 µM) induced similar basophil activation levels to free AX at 680 µM, indicating that **50NpDeAXO** is 23 times more potent in activating basophils from AX-APs than free AX, either by CD63 or CD203c^high^ activation markers.

These results indicate that AX is better recognized and more effective for activation when it is supported on the NpDe compared to the use of free AX. The selected NpDeAXO of 50 nm size interacts with effector cells through more effective multivalent binding of antigenic determinants facilitating the formation of large sIgE-FcεRI clusters, which in turn induce high levels of cell degranulation and serve as a predictive marker of IgE-mediated AX allergy.

### 3.5. Evaluation of the Discrimination Capacity of Allergic Patients and Healthy Controls by **50NpDeAXO**

**Figure 6:**
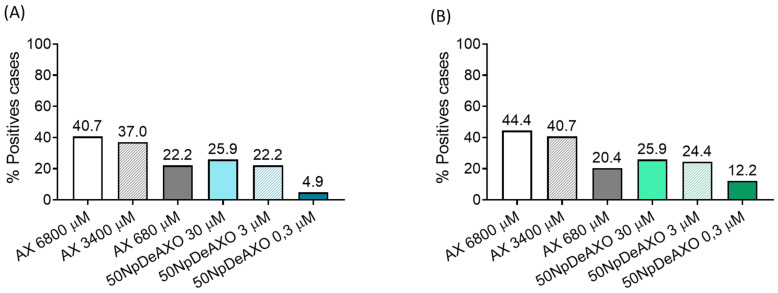
Percentage of positive cases in allergic patients (N = 54) in BAT using CD63 (**A**) and CD203c^high^ (**B**) as basophil activation markers. Positive cases were obtained after using the cut-offs described in Table S4 for AX and **50NpDeAXO** at the different concentrations with both CD63 and CD203c.

To delineate the clinical utility of our Np-based detection system, ROC curves were built to calculate the optimal cut-off and the AUC (Figure S10, Supplementary Materials). Results indicated that **50NpDeAXO** at 30 and 3 µM allowed the detection of 25.9% and 22.2% of positive cases, respectively, through CD63 markers (Figure 6A) and 25.9% and 24.4% at 30 and 3 µM, respectively, through CD203c^high^ markers (Figure 6B). The achieved specificity of NpDeAXO was 94.7% at 30 µM and 95.56% at 3 µM for both markers (Table S4, Supplementary Materials), similar to the values obtained for free AX at 680 µM. The availability of AXO units in the DeAn supported on Nps seems suitable, mimicking its availability in conjugates with natural proteins for recognition by sIgE on basophils and subsequent activation, which would have great potential in clinical application.

## 4. Conclusions

The accurate in vitro diagnosis of BL allergy is a continuing challenge affecting both outpatient and hospital care. By employing a combination of Nps with DeAns instead of the free drug as stimulus, a novel approach to perform the BAT has been developed and studied for first time on human samples. Our results have established that DeAXO-decorated Nps (**NpDeAXO**) of 50 nm in size are effective triggers of basophils from patients with allergy to AX. Moreover, a fully covered surface with DeAXO on Nps is relevant for the spacing between any two AXO moieties to be optimal for the sIgE cross-linking on the basophil surface.

Through this proof-of-concept study, we have demonstrated that **NpDeAXO** Nps are able to induce a more efficient basophil activation than the free AX molecule, as a more than 20-fold lower concentration induces equal or higher levels of basophil activation. This means that AX in the context of the Np system, in comparison with free AX, is better recognized in a more effective way. This indicates that both the availability and the distribution of the AXO antigenic determinants in the dendrimers supported on the Nps is optimal for the efficient immune recognition that takes place in the assay. This demonstrates that Nps can be precise platforms for investigating the suitable size for successful basophil activation and degranulation. Moreover, we have also confirmed that another critical factor in increasing basophil reactivity is the number of AXO units covering the Np. Since the maximum functionalization of the Np surface has already been achieved, the only strategy to increase this hapten density could lie in increasing the number of Nps in the assay (Np concentration). However, the high concentration of Nps affects the stability of the Np dispersions, limiting flow-cytometry measurements. Therefore, further work is needed to overcome the only limitation presented in this study by the nanoparticles: the possibility of increasing the concentration of either Nps or AXO determinants in the assay to achieve more stable dispersions capable of accurate flow-cytometry, which would undoubtedly increase the sensitivity of the test. This work constitutes a starting point for this new approach to BAT-type assays, which, however, requires further optimization of the system for application in clinical practice.

## Figures and Tables

**Table 1:** Chemical properties of the prepared Nps.

Nps	Aspect	Diameter (nm)	AXO (µmolAXO/gNps) ^1^
**20dNpDeAXO**	Suspension	20	290
**30dNpDeAXO**	Suspension	30	270 ^2^
**50dNpDeAXO**	Suspension	50	270 ^2^
**50NpDeAXO**	Solid	50	300
**50Np^0.1De^DeAXO**	Solid	50	130
**50Np^0.01De^DeAXO**	Solid	50	80
**50Np^0.1NH2^DeAXO**	Solid	50	100
**50Np^0.01NH2^DeAXO**	Solid	50	50

^1^ Approximated value from ninhydrin test results (see Tables S1 and S2 of Supplementary Materials). ^2^ No significant differences have been observed between those values.

## Data Availability

Data is contained within the article or Supplementary Material.

## Supplementary Materials

The following supporting information can be downloaded at: https://www.mdpi.com/article/10.3390/pharmaceutics16081039/s1, Figure S1: TEM images and size distribution (determined by DLS; bottom line) of the obtained Nps: (a) **20dNp**; (b) **30dNp**; (c) **50dNp**; (d) **50Np**; Figure S2: FSC vs. SSC FACS dot plots of samples treated with free AX at 6800, 3400, and 680 µM and **50NpDeAXO** at 300, 30, and 3 µM of AXO. The debris smear observed at the highest concentration of Nps tested is depicted by a red ellipse. NS: non-stimulated, fMLP: N-Formylmethionyl-leucyl-phenylalanine, aIgE: anti-immunoglobulin E antibody; Figure S3. Viability of AX and **50NpDeAXO**-treated basophils. HC and AP samples were treated with free AX or **50NpDeAXO** at the concentrations used in the BAT and stained with L/D viability fluorescent dye and analyzed by FACS. (**A**) Representative plots of gating strategy and analysis. (B) % Viability of the low signal region of the plots (mean +/− SD). NS: non-stimulated, fMLP: N-Formylmethionyl-leucyl-phenylalanine, aIgE: anti-immunoglobulin E antibody; Figure S4: CD63 and CD203c^high^ ROC curves of free AX and **50NpDeAXO** at three different concentrations; Figure S5: DLS spectrum of a dispersion of **50NpDeAXO** 3 hours after its preparation; Figure S6: DLS spectrum of a dispersion of **50NpDeAXO** 4 hours after its preparation; Figure S7: DLS spectrum of a dispersion of **50NpDeAXO** 5 hours after its preparation; Figure S8: FSC *vs* SSC FACS dot plots of samples treated with free AX at 6800, 3400 and 680 μM and **50NpDeAXO** at at 300, 30 and 3 μM of AXO. The debris smear observed at the highest concentration of Nps tested is depicted by a red ellipse. NS: non-stimulated, fMLP: N-Formylmethionyl-leucyl-phenylalanine, aIgE: anti-Immunoglobulin E antibody; Figure S9: Viability of AX and **50NpDeAXO**-treated basophils. HC and AP samples were treated with free AX or **50NpDeAXO** at the concentrations used in the BAT and stained with L/D viability fluorescent dye, and analysed by FACS. A) representative plots of gating strategy and analysis, B) % Viability of the low signal region of the plots (mean +/- SD). NS: non-stimulated, fMLP: N-Formylmethionyl-leucyl-phenylalanine, aIgE: anti-Immunoglobulin E antibody; Figure S10: CD63 and CD203chigh ROC curves of free AX and **50NpDeAXO** at three different concentrations; Table S1: Chemical properties of the prepared Nps in dispersion; Table S2: Chemical properties of the prepared solid Nps; Table S3: Characteristics of the AX-allergic cohort; Table S4: AUC, cut-off, sensitivity, specificity with the corresponding 95% CI and the *p*-value of (**A**) free AX and (B) 50NpDeAXO at three different concentrations, using CD63 or CD203^c^high basophil activation markers. * *p* < 0.05, ** *p* < 0.01.
