# Supplementary material for: New Approaches for Basophil Activation Tests Employing Dendrimeric Antigen–Silica Nanoparticle Composites
Source: Pharmaceutics. 2024 Aug 3;16(8):1039. doi: 10.3390/pharmaceutics16081039 (PMC11359297; doi:10.3390/pharmaceutics16081039)
Supplement: Supplementary file 1 [file pharmaceutics-16-01039-s001.zip › pharmaceutics-3117396-supplementary.pdf]

Electronic Supporting Information

New approaches for Basophil Activation Tests employing Dendrimeric Antigen-Silica Nanoparticle composites

Silvia Calvo-Serrano[1,2,†], Esther Matamoros[3,4,†], Jose Antonio Céspedes[1], Rubén Fernández-Santamaría[1], Violeta Gil-Ocaña[3,4], Ezequiel Perez-Inestrosa[3,4], Cecilia Frecha[1], Maria I. Montañez[1,3,*], Yolanda Vida[3,4,*], Cristobalina Mayorga[1,5,‡], Maria J. Torres[1,2,5,‡]

[1] Allergy Research Group, Instituto de Investigación Biomédica de Málaga y Plataforma en Nanomedicina–IBIMA Plataforma Bionand. Parque Tecnológico de Andalucía, 29590 Málaga, Spain; RICORS Red de Enfermedades Inflamatorias (REI), Madrid, Spain; silcalser@gmail.com (S.C.); jcespedeslagos13@gmail.com (J.A.C.); rubenfernandezsantamaria@gmail.com (R.F.S.); frechacecilia@gmail.com (C.F.); maribelmv@uma.es (M.I.M); lina.mayorga@ibima.eu (C.M.); mjtorresj@uma.es (M.J.T.).

[2] Departamento de Medicina y Dermatología, Universidad de Málaga, Málaga, Spain; silcalser@gmail.com (S.C.); mjtorresj@uma.es (M.J.T.).

[3] Departamento de Química Orgánica, Universidad de Málaga, Campus Teatinos s/n, 29071 Málaga, Spain; vgil51192@gmail.com (V.G.); esthermc@uma.es (E.M.); maribelmv@uma.es (M.I.M); yolanda.vida@uma.es (Y.V.); inestrosa@uma.es (E.P.I.)

[4] Instituto de Investigación Biomédica de Málaga y Plataforma en Nanomedicina – IBIMA Plataforma Bionand, Parque Tecnológico de Andalucía, 29590 Málaga, Spain; vgil51192@gmail.com (V.G.); esthermc@uma.es (E.M.); yolanda.vida@uma.es (Y.V.); inestrosa@uma.es (E.P.I.)

[5] Allergy Unit, Hospital Regional Universitario de Málaga-HRUM, Málaga, Spain; lina.mayorga@ibima.eu (C.M.); mjtorresj@uma.es (M.J.T.).

* Correspondence: yolanda.vida@uma.es (Y.V.); maribelmv@uma.es (M.I.M)

† These authors contributed equally to this work and share first authorship

‡ These authors contributed equally to this work and share last authorship

Table of Contents

Nps surface modification **2**
Figure S1. 2
Nps surface stability **3**
Figure S2 **3**
Figure S3. 3
Figure S4. 3
Figure S5. 4
Figure S6. 4
Figure S7. 4
Figure S8. 5
Figure S9. 6
Figure S10. 7
Table S1. 8
Table S2. 8
Table S3. 9
Table S4. 12

Nps surface modification

- **Nps amino functionalization**

*General procedure for **50Np***. Employed amounts of APTES per 100 mg of **50NP**: 1.67 mmol to obtain **50NpNH$_2$**; 0.167 mmol to obtain **50Np$^{0.1}$NH$_2$**; and 0.0167 mmol to obtain **50Np$^{0.01}$NH$_2$**.

- **Nps carboxy functionalization**

General procedure for **50NpNH$_2$**, **50Np$^{0.1}$NH$_2$** and **50Np$^{0.01}$NH$_2$**. Employed amounts: 2.25 mmol of succinic anhydride and $2.4 \cdot 10^{-1}$ mmol of TEA per 100 mg of **50NpNH$_2$** to obtain **50NpCO$_2$H**; $2.25 \cdot 10^{-1}$ mmol of succinic anhydride and $2.4 \cdot 10^{-2}$ mmol of TEA per 100 mg of **50Np$^{0.1}$NH$_2$** to obtain **50Np$^{0.1NH2}$CO$_2$H**; $2.25 \cdot 10^{-2}$ mmol of succinic anhydride and $2.4 \cdot 10^{-3}$ mmol of TEA per 100 mg of **50Np$^{0.01}$NH$_2$** to obtain **50Np$^{0.01NH2}$CO$_2$H**.

- **Nps dendrimer functionalization**

General procedure for **50NpCO$_2$H**, **50Np$^{0.1NH2}$CO$_2$H** and **50Np$^{0.01NH2}$CO$_2$H.** Employed amounts: $1.8 \cdot 10^{-1}$ mmol of EDCI, $3.5 \cdot 10^{-1}$ mmol of NHS and $5.2 \cdot 10^{-3}$ mmol of PAMAM-G2 per 100 mg of **50NpCO$_2$H** to obtain **50NpDe**; $1.8 \cdot 10^{-2}$ mmol of EDCI, $3.5 \cdot 10^{-2}$ mmol of NHS and $5.2 \cdot 10^{-4}$ mmol of PAMAM-G2 per 100 mg of **50NpCO$_2$H** to obtain **50Np$^{0.1}$De**; $1.8 \cdot 10^{-3}$ mmol of EDCI, $3.5 \cdot 10^{-3}$ mmol of NHS and $5.2 \cdot 10^{-5}$ mmol of PAMAM-G2 per 100 mg of **50NpCO$_2$H** to obtain **50Np$^{0.01}$De**; $1.8 \cdot 10^{-2}$ mmol of EDCI, $3.5 \cdot 10^{-2}$ mmol of NHS and $5.2 \cdot 10^{-4}$ mmol of PAMAM-G2 per 100 mg of **50Np$^{0.1NH2}$CO$_2$H** to obtain **50Np$^{0.1NH2}$De**; $1.8 \cdot 10^{-3}$ mmol of EDCI, $3.5 \cdot 10^{-3}$ mmol of NHS and $5.2 \cdot 10^{-5}$ mmol of PAMAM-G2 per 100 mg of **50Np$^{0.01NH2}$CO$_2$H** to obtain **50Np$^{0.01NH2}$De**.

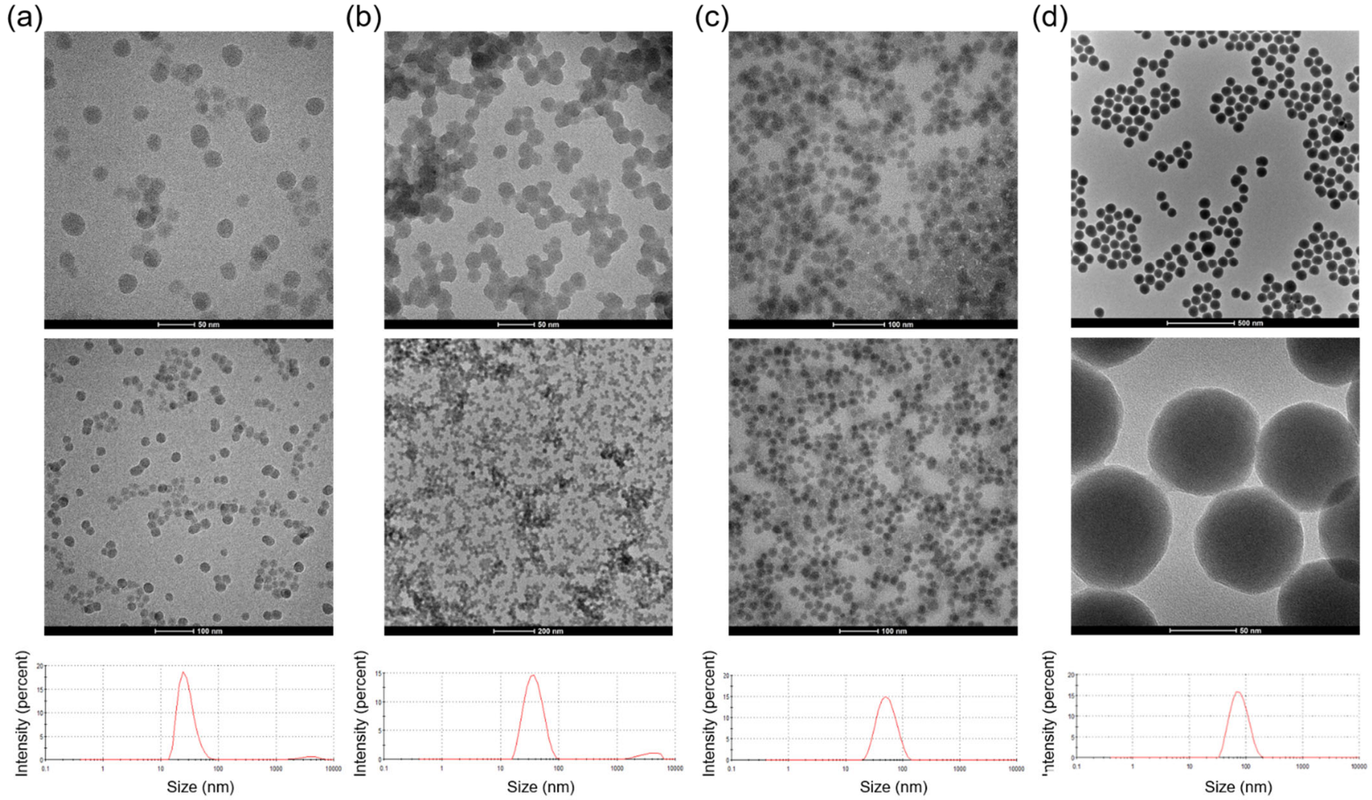

Figure S1. TEM images and size distribution (determined by DLS; bottom line) of the obtained Nps (a) **20dNp**; (b) **30dNp**; (c) **50dNp**; (d) **50Np**.

Nps stability

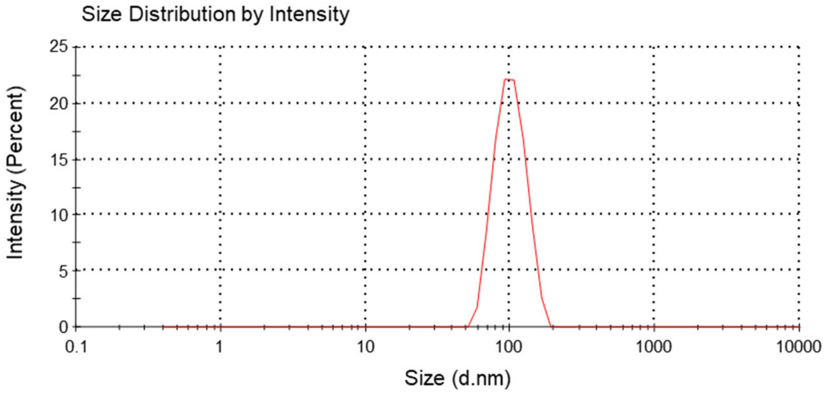

Figure S2. DLS spectrum of a freshly prepared dispersion of **50NpDeAXO** (after storage for about six months as a solid at 5ºC).

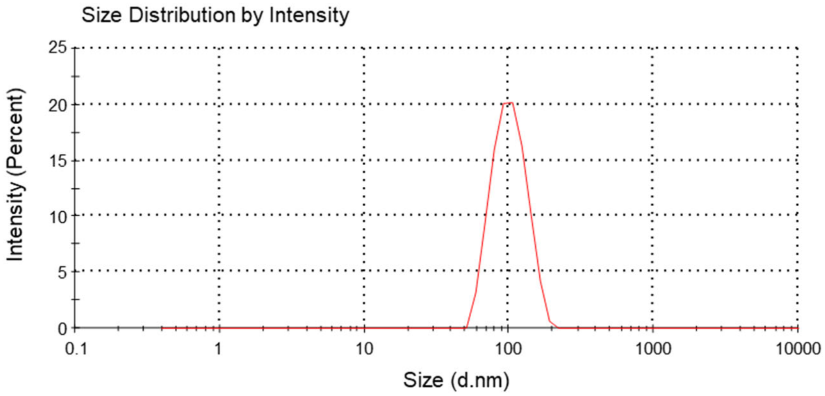

Figure S3. DLS spectrum of a dispersion of **50NpDeAXO** 1 hours after its preparation.

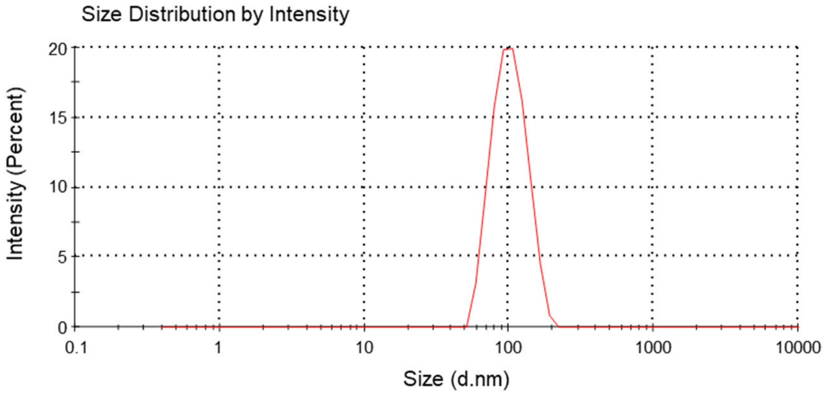

Figure S4. DLS spectrum of a dispersion of **50NpDeAXO** 2 hours after its preparation.

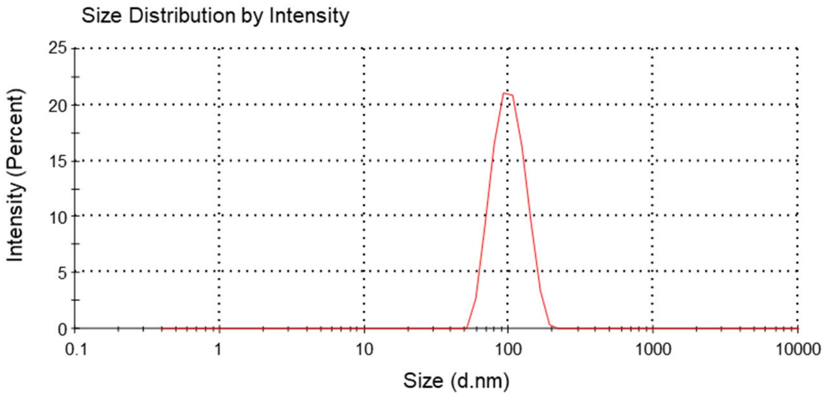

Figure S5. DLS spectrum of a dispersion of **50NpDeAXO** 3 hours after its preparation.

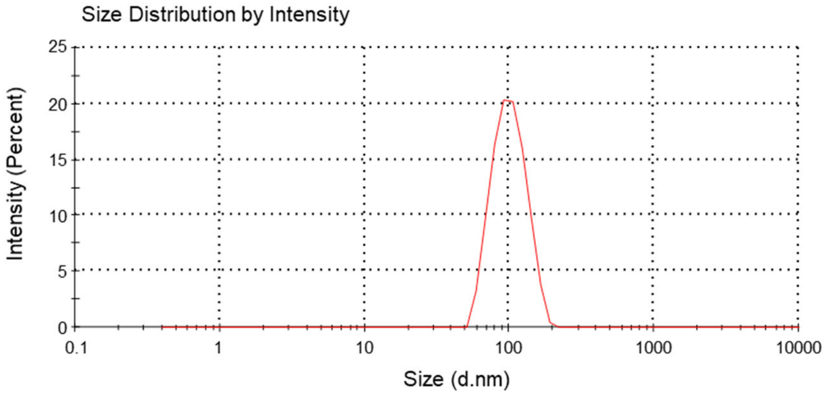

Figure S6. DLS spectrum of a dispersion of **50NpDeAXO** 4 hours after its preparation.

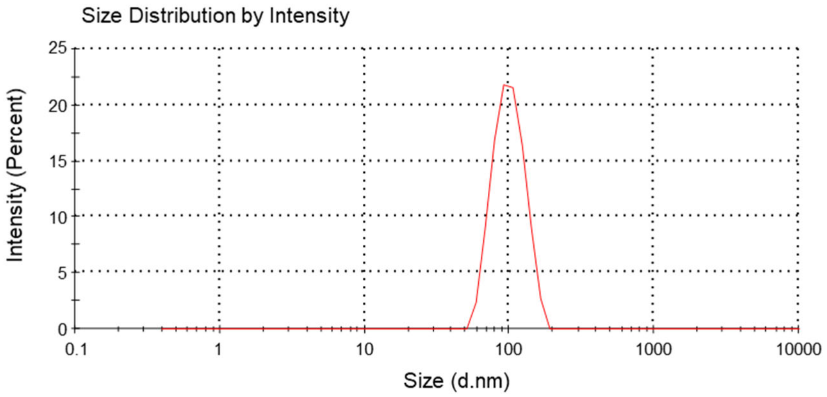

Figure S7. DLS spectrum of a dispersion of **50NpDeAXO** 5 hours after its preparation.

All spectra were recorded using a Malvern Zetasizer Nano ZS90 instrument in aqueous solutions. Measurement obtained directly from the instrument are attached (as pdf reports).

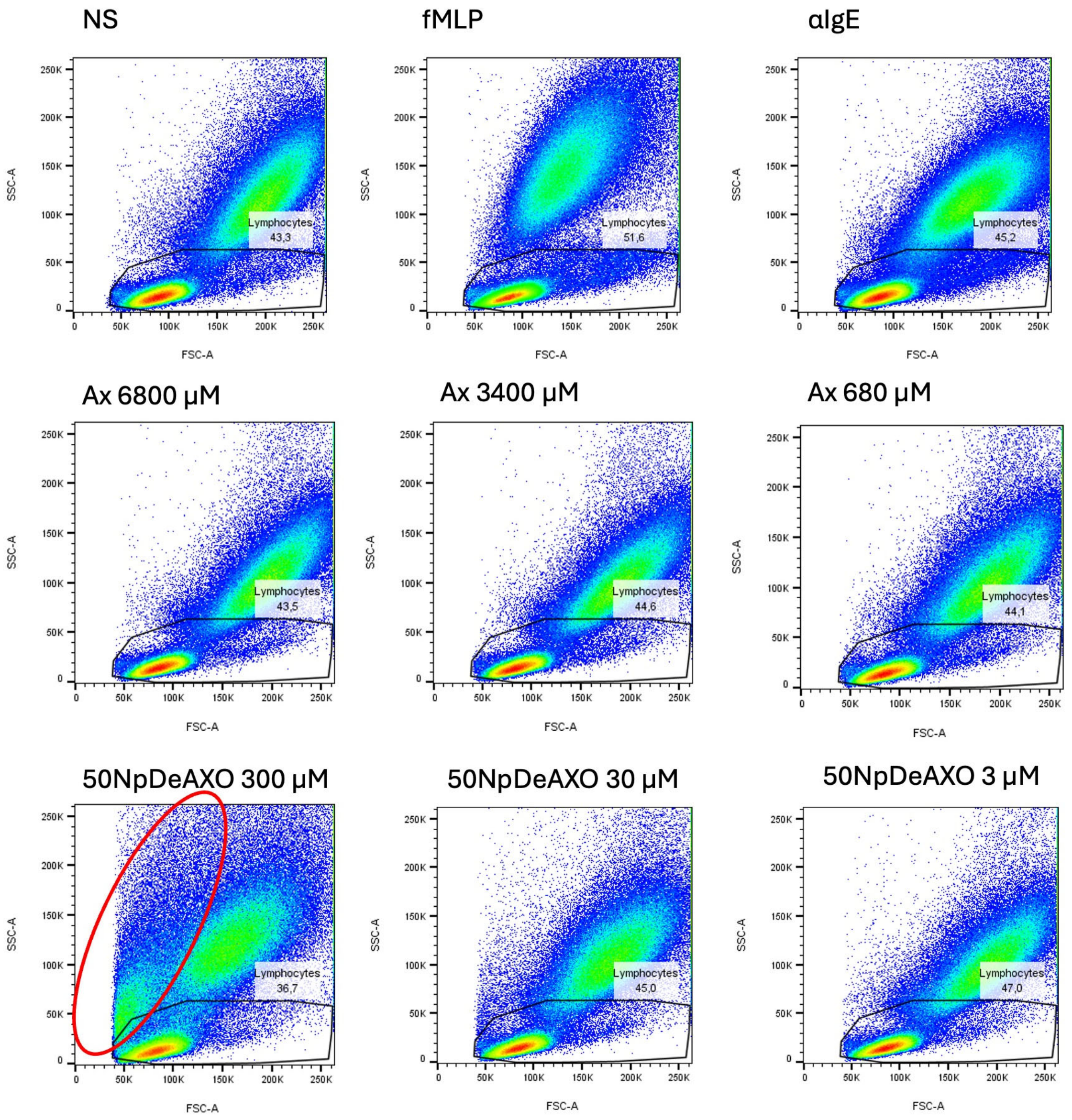

Figure S8. FSC *vs* SSC FACS dot plots of samples treated with free AX at 6800, 3400 and 680 μM and **50NpDeAXO** at at 300, 30 and 3 μM of AXO. The debris smear observed at the highest concentration of Nps tested is depicted by a red ellipse. NS: non-stimulated, fMLP: N-Formylmethionyl-leucyl-phenylalanine, aIgE: anti-Immunoglobulin E antibody.

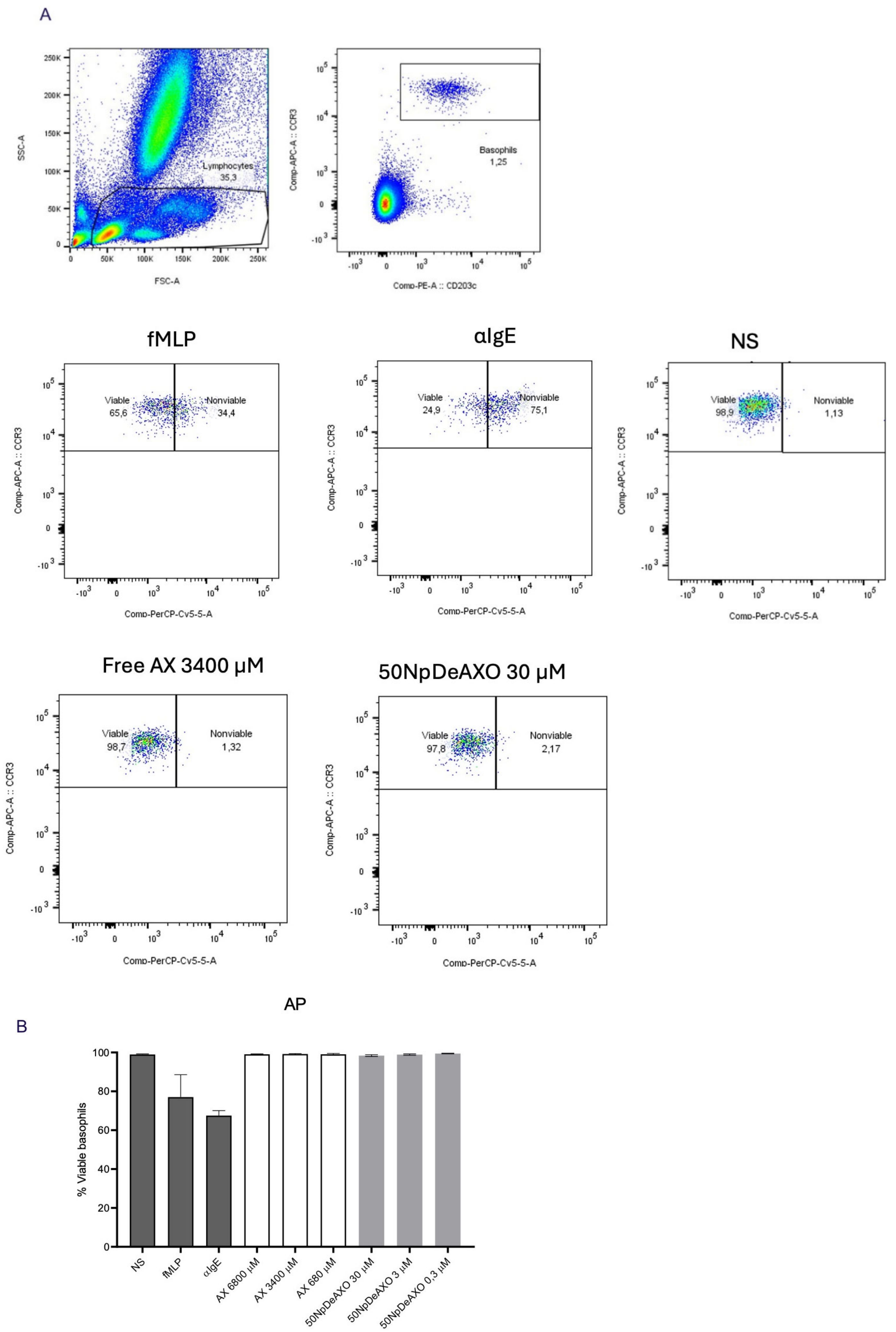

Figure S9. Viability of AX and **50NpDeAXO**-treated basophils. HC and AP samples were treated with free AX or **50NpDeAXO** at the concentrations used in the BAT and stained with L/D viability fluorescent dye, and analysed by

FACS. A) representative plots of gating strategy and analysis, B) % Viability of the low signal region of the plots (mean +/- SD). NS: non-stimulated, fMLP: N-Formylmethionyl-leucyl-phenylalanine, aIgE: anti-Immunoglobulin E antibody.

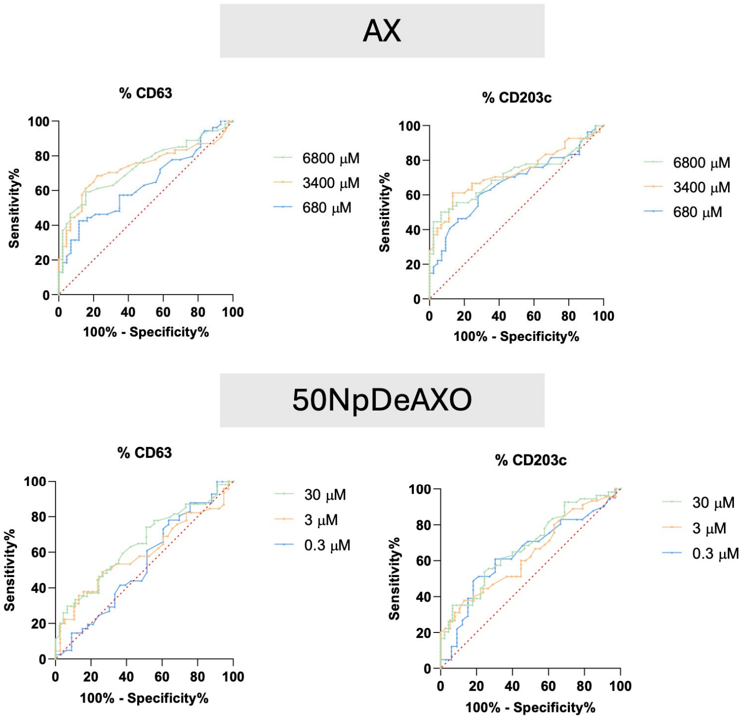

Figure S10. CD63 and CD203c^{high} ROC curves of free AX and **50NpDeAXO** at three different concentrations.

Table S1. Chemical properties of the prepared Nps in dispersion.

**Nps**	**20dNp**	**20dNpNH₂**	**20dNpCO₂H**	**20dNpDe**	**20dNpDeAXO**
**ξ (mV)**	-38 ± 4	20 ± 3	-25 ± 3	43 ± 6	-7 ± 2
**µmol NH_2/gNps**	--	200	65	287	--
**Nps**	**30dNp**	**30dNpNH_2**	**30dNpCO_2H**	**30dNpDe**	**30dNpDeAXO**
**ξ (mV)**	-31 ± 6	25 ± 5	-22 ± 5	42 ±6	-9 ±4
**µmol NH_2/gNps**	--	200	58	274	--
**Nps**	**50dNp**	**50dNpNH_2**	**50dNpCO_2H**	**50dNpDe**	**50dNpDeAXO**
**ξ (mV)**	-38 ± 4	22 ± 4	-29 ± 4	42 ± 5	-9± 3
**µmol NH_2/gNps**	--	194	66	270	--
**Terminal group**	**OH**	**NH_2**	**CO_2H**	**NH_2**	**AXO**

Table S2. Chemical properties of the prepared solid Nps.

**Nps**	**50dNp**	**50dNpNH_2**	**50dNCO_2H**	**50dNDe**	**50dNpDeAXO**
**ξ (mV)**	-31 ±5	29 ±4	-29 ±5	30 ±4	-10 ±5
**µmol NH_2/gNps**	--	209	47	295	--
**Nps**				**50Np$^{0.1}$De**	**50Np$^{0.1De}$DeAXO**
**ξ (mV)**				27 ±10	-23±13
**µmol NH_2/gNps**				84	--
**Nps**				**50Np$^{0.01}$De**	**50Np$^{0.01De}$DeAXO**
**ξ (mV)**				-17 ±3	-26±11
**µmol NH_2/gNps**				52	--
**Nps**		**50Np$^{0.1}$$NH_2$**	**50Np$^{0.1NH2}$$CO_2$H**	**50Np$^{0.1NH2}$De**	**50Np$^{0.1NH2}$DeAXO**
**ξ (mV)**		19 ±6	-25 ±4	26 ±10	-29±13
**µmol NH_2/gNps**		151	30	69	--
**Nps**		**50Np$^{0.01}$$NH_2$**	**50Np$^{0.01NH2}$$CO_2$H**	**50Np$^{0.01NH2}$De**	**50Np$^{0.01NH2}$DeAXO**
**ξ (mV)**		16 ±6	-20 ±3	4 ±10	-34±10
**µmol NH_2/gNps**		97	25	38	--
**Terminal group**	**OH**	**NH_2**	**CO_2H**	**NH_2**	**AXO**

Table S3. Characteristics of the AX-allergic cohort.

Patient	Sex	Age	Drug involved	Latency (min)	Reaction grade	Skin Test								DPT
						SPT				IDT				
						BP-OL	MD	AX	CLV	BP-OL	MDM	AX	CLV	AX
P1	M	25	AX-CLV	10	II	-	-	-	-	-	-	+	-	ND
P2	F	37	AX-CLV, AX	30-15	I	-	-	-	-	-	-	-	-	ND[§]
P3	M	54	AX	2	III	-	-	+	ND	-	-	-	ND	ND
P4	F	66	AX	<60	II	-	-	-	ND	-	-	+	ND	ND
P5	M	44	UK	<5	II	-	-	-	-	-	-	+	-	ND
P6	F	39	Meropenem	<10	III	-	-	-	-	-	-	-	-	ND[§]
P7	F	67	AX	<60	II	-	-	-	ND	-	-	+	ND	ND
P8	F	57	AX-CLV	10	II	-	-	-	-	-	-	+	-	ND
P9	M	61	AX-CLV	10	I	-	-	+	-	-	-	+	-	ND
P10	F	36	AX-CLV	>360	II	-	-	+	-	-	-	-	-	ND
P11	F	UK	AX	UK	II	-	-	-	-	-	-	-	-	ND
P12	F	50	AX-CLV, AX	UK	II	-	-	-	-	-	-	+	-	ND
P13	M	34	AX-CLV	UK	II	-	-	-	-	-	-	-	+	+
P14	M	26	AX-CLV	10	II	-	-	-	-	-	-	+	-	ND
P15	F	43	AX-CLV	<60	II	-	-	-	-	-	-	+	-	ND
P16	M	32	AX-CLV	15	II	-	-	-	-	-	-	+	-	ND
P17	M	35	AX-CLV	30-45	II	-	-	-	-	-	-	-	-	ND[§]
P18	F	56	AX-CLV	60-360	I	-	-	-	-	-	-	+	-	ND
P19	M	69	AX-CLV	UK	III	-	-	-	-	-	-	+	-	ND*
P20	M	39	AX-CLV	UK	II	-	-	-	-	-	-	+	-	ND
P21	F	77	AX	45	II	-	-	-	-	-	-	+	-	ND
P22	M	29	AX, AX-CLV	10-15	II	-	-	-	-	-	-	+	-	ND
P23	F	50	AX-CLV	30	II	-	-	-	-	-	-	+	-	ND
P24	F	17	AX-CLV	120-180	II	-	-	-	-	-	-	+	+	ND
P25	F	70	AX-CLV	120-180	II	-	-	-	-	-	-	+	+	ND
P26	F	57	AX-CLV	15	I	-	-	-	-	-	-	+	-	ND

P27	M	35	AX-CLV	UK	II	-	-	-	-	-	-	-	+	+
P28	F	48	AX	60	I	-	-	-	ND	-	-	+	ND	ND
P29	F	30	AX	30	II	-	-	+	ND	-	-	-	ND	ND
P30	M	62	AX	<1	III	-	-	ND	ND	-	-	ND	ND	ND§
P31	F	71	AX-CLV	90-120	I	-	-	-	ND	-	-	+	-	ND
P32	F	74	Penicillin	45/90	II	-	-	-	-	-	-	-	-	ND*
P33	M	63	AX	5	II	-	-	-	ND	-	-	-	ND	ND*
P34	M	62	AX	<1	III	-	-	ND	ND	-	-	ND	ND	ND§
P35	F	71	AX-CLV	90-120	II	-	-	-	-	-	-	+	-	ND
P36	F	21	AX-CLV	30	II	-	-	-	-	-	-	+	-	ND
P37	F	47	AX-CLV	20	II	-	-	-	-	-	-	+	-	ND
P38	F	65	AX-CLV	<30	II	-	-	-	-	-	-	-	-	ND *
P39	F	57	AX-CLV	10	I	-	-	-	-	-	-	+	-	ND
P40	F	42	AX-CLV	120-180	II	-	-	-	-	-	-	-	+	ND
P41	F	38	AX-CLV	2	II	-	-	+	-	-	-	-	-	ND
P42	F	59	AX-CLV	10	II	-	-	-	-	-	-	+	-	ND
P43	F	72	AX-CLV	90-120	I	-	-	-	-	-	-	+	-	ND
P44	F	39	AX-CLV	5	II	-	-	-	-	-	-	+	-	ND
P45	F	19	AX-CLV	UK	II	-	-	+	-	-	-	+	-	ND
P46	F	46	AX	20	II	-	-	-	-	-	-	+	ND	ND
P47	F	71	Penicillin V/Cefuroxime	UK	I	-	-	-	-	-	-	-	-	ND*
P48	F	32	AX-CLV	30-45	II	-	-	-	-	-	-	-	+	ND
P49	F	69	AX	<60	II	-	-	-	-	-	-	+	-	ND
P50	F	67	AX-CLV	<60	II	-	-	-	-	-	-	+	-	ND
P51	UK	UK	AX	UK	II	-	-	-	-	-	-	+	-	ND
P52	F	46	AX/Cloxacillin	15	II	-	-	-	-	-	-	-	-	ND§
P53	F	66	AX	5	I	-	-	-	-	-	-	+	-	ND
P54	F	77	AX-CLV	10	III	-	-	-	-	-	-	-	-	ND*

P55	F	35	AX	5	II	-	-	-	-	-	-	+	-	ND
P56	F	41	AX	30-45	II	-	-	+	-	-	-	-	-	ND
P57	M	32	AX-CLV, AX	20	I	-	-	-	-	-	-	+	-	ND
P58	M	62	AX	1	II	-	-	-	-	-	-	+	-	ND
P59	F	67	AX	< 5	II	-	-	-	-	-	-	+	-	ND
P60	M	62	AX	1	II	-	-	+	-	-	-	-	-	ND
P61	F	76	AX	UK	I	-	-	+	-	-	-	-	-	ND
P62	M	51	AX-CLV	<60	I	-	-	+	-	-	-	-	-	ND
P63	F	61	AX-CLV	60-360	I	-	-	-	-	-	-	+	-	ND
P64	F	54	AX	15	III	-	-	+	-	-	-	-	-	ND
P65	F	67	AX-CLV	UK	II	-	-	-	-	-	-	-	+	ND*
P66	F	36	AX	180	II	-	-	-	-	-	-	-	-	ND*
P67	M	54	AX	10	III	-	-	+	-	-	-	-	-	ND
P68	M	86	Penicillin	120	II	-	-	-	-	-	-	+	-	ND
P69	M	51	AX	15-20	II	-	-	-	-	-	-	+	-	ND
P70	M	46	AX	3	II	-	-	-	-	-	-	+	-	ND

Clinical characteristics of the allergic cohort. F: female, M: male, Latency (min): time interval between challenge and reaction development, AX: amoxicillin, CLV: clavulanic acid, BP-OL: benzylpenicilloyl-octa-L-lysine, MD: minor determinant (sodium benzylpenilloate), SPT: skin prick test, IDT: intradermal test, DPT: drug provocation test, ND: not done, (*) presence of comorbidities, (§) high severity of symptoms.

Table S4. AUC, Cut-off, Sensitivity, Specificity with the corresponding 95% CI and the p-value of (A) free AX and (B) **50NpDeAXO** at three different concentrations, using CD63 or CD203c^{high} basophil activation markers. * $p<0.05$, ** $p<0.01$.

(A)

AX

AXO	AUC	Cut-off	Sensitivity (%)	95% CI	Specificity (%)	95% CI	p-value
**% CD63**							
**680 µM**	0.6380	7.55	22.22	(13.20, 34.94)	95.35	(84.54, 99.17)	*
**3400 µM**	0.7323	4.75	37.04	(25.42, 50.37)	95.56	(85.17, 99.21)	****
**6800 µM**	0.7366	3.90	40.74	(28.68, 54.03)	95.56	(85.17, 99.21)	****
**% CD203c**							
**680 µM**	0.6665	8.35	22.22	(13.20, 34.94)	95.35	(84.54, 99.17)	**
**3400 µM**	0.7311	5.10	40.74	(28.68, 54.03)	95.56	(85.17, 99.21)	****
**6800 µM**	0.7123	4.75	44.44	(32.00, 57.62)	95.56	(85.17, 99.21)	***

(B)

50NpDeAXO

NP-AXO	AUC	Cut-off	Sensitivity (%)	95% CI	Specificity (%)	95% CI	p-value
**% CD63**							
**0.3 µM**	0.5347	10.35	4.878	(0.8667, 16.14)	93.94	(80.39, 98.92)	ns
**3.0 µM**	0.5915	7.4	22.22	(12.54, 36.27)	94.74	(82.71, 99.06)	ns
**30 µM**	0.6463	9.75	25.93	(16.12, 38.93)	95.56	(85.17, 99.21)	*
**% CD203c**							
**0.3 µM**	0.6397	9.05	12.20	(5.323, 25.54)	93.94	(80.39, 98.92)	*
**3.0 µM**	0.6371	7.05	26.67	(15.96, 41.04)	94.74	(82.71, 99.06)	*
**30 µM**	0.6829	13.10	25.93	(16.12, 38.93)	95.56	(85.17, 99.21)	**
